# The biology and type I/III hybrid nature of type I-D CRISPR–Cas systems

**DOI:** 10.1042/BCJ20220073

**Published:** 2023-04-13

**Authors:** Tess M. McBride, Shaharn C. Cameron, Peter C. Fineran, Robert D. Fagerlund

**Affiliations:** 1 Department of Microbiology and Immunology, University of Otago, PO Box 56, Dunedin 9054, New Zealand; 2 Genetics Otago, University of Otago, Dunedin, New Zealand; 3 Bioprotection Aotearoa, University of Otago, PO Box 56, Dunedin 9054, New Zealand

**Keywords:** bacteriophages, CRISPR, protein structure, RNA-binding proteins

## Abstract

Prokaryotes have adaptive defence mechanisms that protect them from mobile genetic elements and viral infection. One defence mechanism is called CRISPR–Cas (clustered regularly interspaced short palindromic repeats and CRISPR-associated proteins). There are six different types of CRISPR–Cas systems and multiple subtypes that vary in composition and mode of action. Type I and III CRISPR–Cas systems utilise multi-protein complexes, which differ in structure, nucleic acid binding and cleaving preference. The type I-D system is a chimera of type I and III systems. Recently, there has been a burst of research on the type I-D CRISPR–Cas system. Here, we review the mechanism, evolution and biotechnological applications of the type I-D CRISPR–Cas system.

## Introduction

Prokaryotes are under constant predation by their viruses and mobile genetic elements (MGEs), such as plasmids [1]. To counteract infection, prokaryotes have evolved an arsenal of diverse defence mechanisms [2]. CRISPR–Cas (clustered regularly interspaced short palindromic repeats and CRISPR-associated proteins) systems are heritable, adaptive immune systems that are present in ∼80% and ∼40% of all sequenced archaeal and bacterial genomes, respectively [2–5]. The loci of CRISPR–Cas systems consist of CRISPR arrays, which store foreign nucleic acid sequences from previous infections, and *cas* genes that encode the structural and catalytic proteins of the system. When re-exposed to an MGE, the invading nucleic acids are sequence-specifically targeted and degraded by CRISPR–Cas complexes to eliminate the threat.

**Figure 1.**
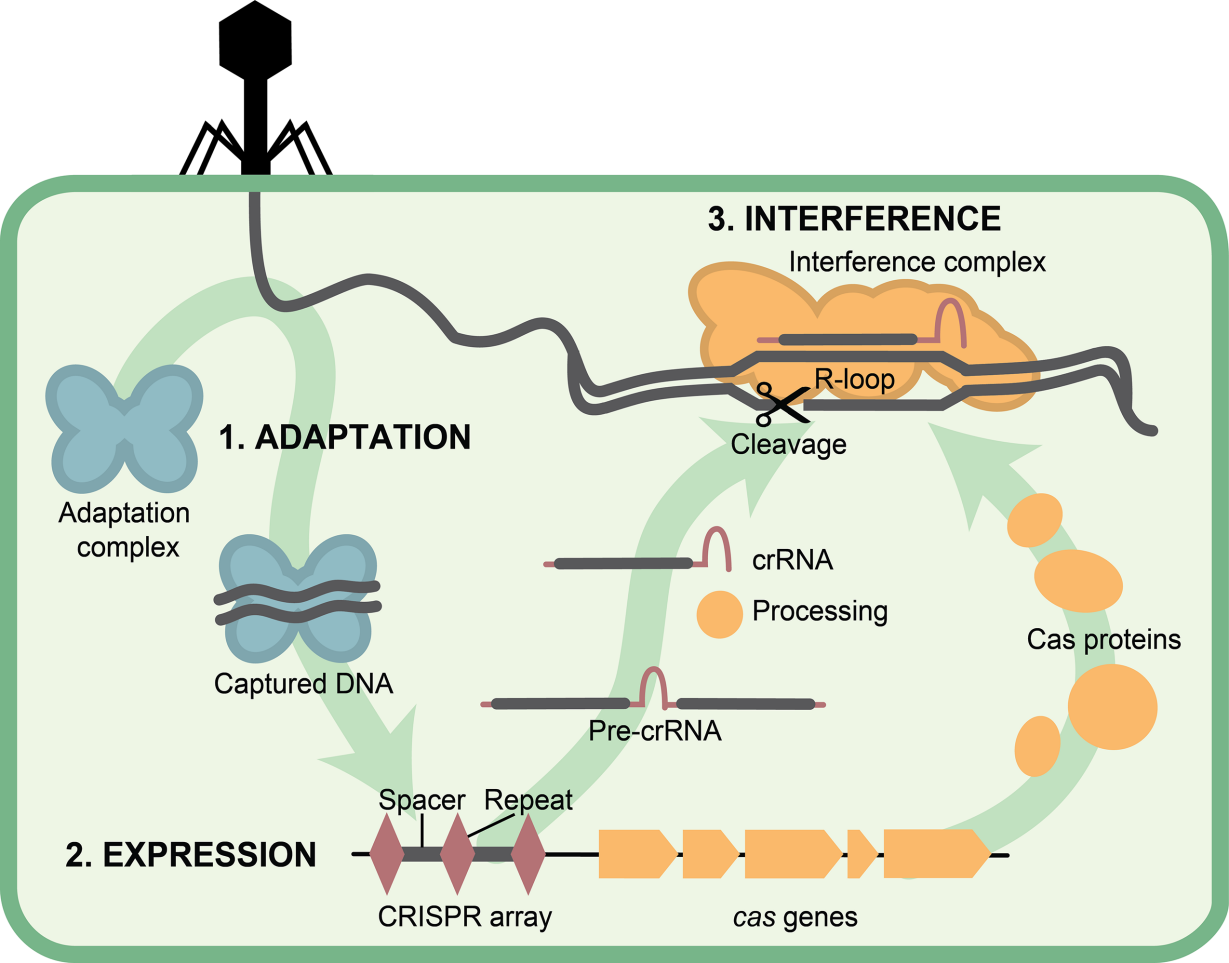
CRISPR–Cas stages of defence. Adaptation occurs when the adaptation complex (blue) captures DNA, which it then incorporates into the CRISPR array as a spacer (grey line). The expression stage occurs when the *cas* genes (yellow) are expressed, and the CRISPR array is transcribed into pre-crRNA, which is subsequently processed by a Cas protein into crRNA. Interference occurs when the Cas proteins and crRNA assemble into the interference complex and then binds to complementary DNA and degrades it. Stages of defence based on type I CRISPR–Cas systems.

There are three stages of CRISPR–Cas immunity: adaptation, expression and processing, and interference (Figure 1) [6]. The core adaptation machinery, Cas1 and Cas2, are conserved throughout almost all CRISPR–Cas systems [7]. During adaptation, Cas1 and Cas2 associate to form an adaptation complex that captures a foreign nucleic acid fragment, a prespacer, and incorporates it into the CRISPR array [8–10]. During expression and processing, the *cas* genes are transcribed and translated, while the CRISPR array is transcribed into one long RNA known as precursor-CRISPR RNA (pre-crRNA). The pre-crRNA is processed by an endoribonuclease, which generates a shorter crRNA [11–13]. During interference, the Cas protein(s) and the crRNA form an interference complex [14]. This interference complex uses the crRNA as a guide to bind the complementary invading nucleic acid target known as a ‘protospacer’. Once the complex has bound the target, Cas protein(s) degrade the invading nucleic acid(s), protecting the host cell [15].

CRISPR–Cas systems have a wide diversity of structural and mechanistic attributes and are classified into two classes, six types, and over 30 subtypes [16]. Class 1 CRISPR–Cas systems are characterised by their multi-protein interference complexes and account for ∼90% of all sequenced and classified CRISPR–Cas systems, while class 2 systems are defined by having a single interference protein [4]. Class 1 systems can be further divided into types I, III, and IV based on their ‘signature’ genes.

**Figure 2.**
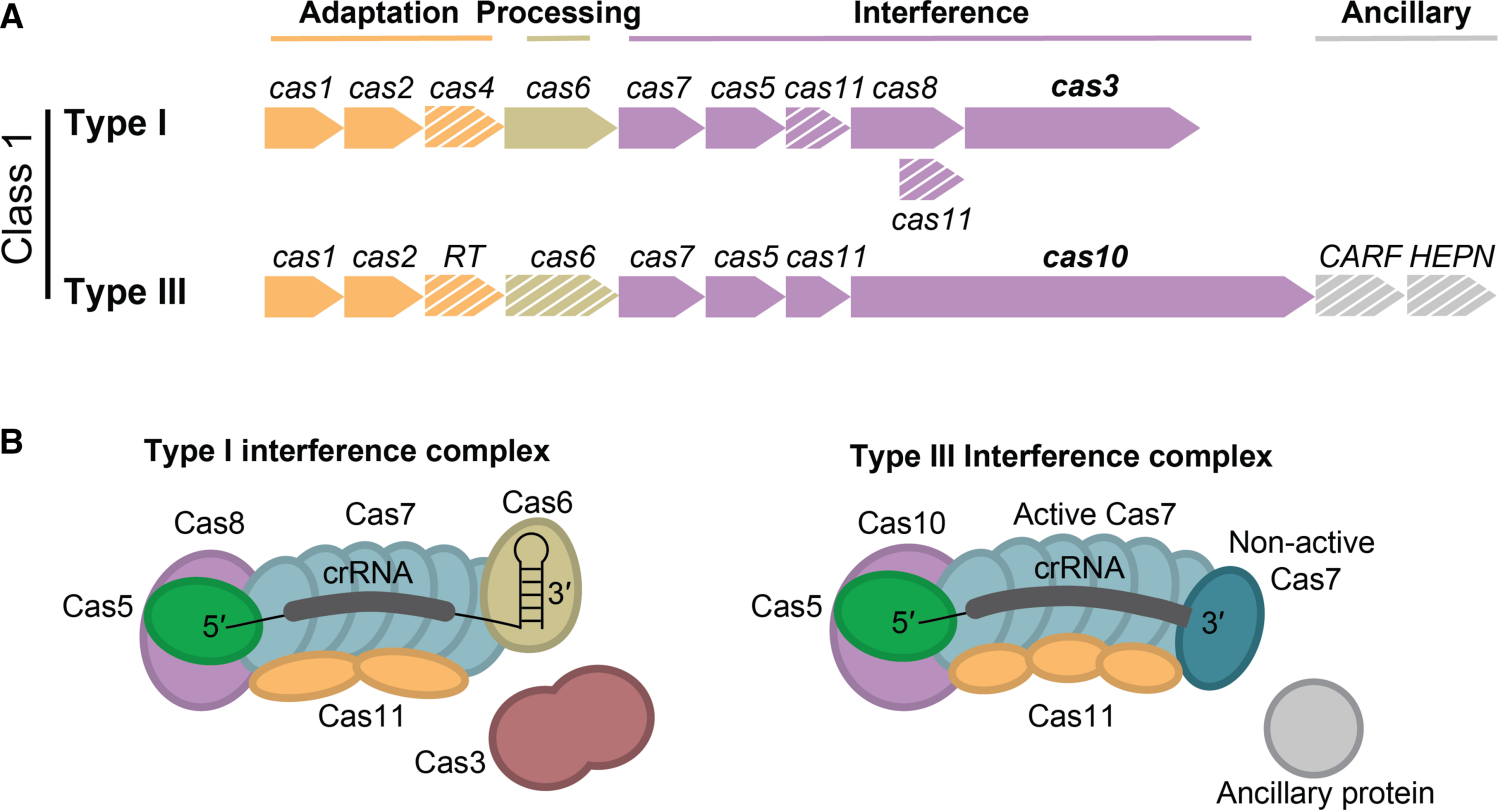
Classification Type I and type III CRISPR–Cas systems. (**A**) Comparison of type I and III CRISPR–Cas systems, with their ‘signature’ genes in bold. Genes are organised by role; adaptation (yellow), processing (olive green), interference (purple), and ancillary (grey). Genes absent from some subtypes are indicated with white diagonal lines. Diagram adapted from Makarova et al. [16]. (**B**) Schematic of type I and type III interference complexes with proteins annotated.

This review focuses on the type I-D CRISPR–Cas system, which is a hybrid of type I and III systems (Figure 2A) [7]. Early bioinformatic evidence indicated the type I-D system was likely an evolutionary ancestor of class 1 systems, linking a type III-like ancestor to the typical type I system [7]. Recently, the mechanism of type I-D immunity has begun to be discovered, revealing structural and mechanistic features of both type I and type III systems [7,17–19]. After the burst of research into class 2 complexes as genetic editing tools, the spotlight has started to fall on class 1 systems to expand the CRISPR toolbox [20]. Indeed, type I-D Cascade shows potential for novel biotechnological applications [21–23].

## Class 1 systems

The class 1 type I and III systems have a common evolutionary lineage and share a similar interference complex architecture [4,24]. The interference complexes typically have a major filament comprised of multiple Cas7 (Csm3/Cmr4 in type III) subunits that bind along the length of the crRNA in a helical fashion (Figure 2B). The 3′ end of the crRNA can be capped with Cas6 in type I systems or with a non-catalytic Cas7 (Csm5/Cmr6) in type III systems. The 5′ end of the crRNA is capped with Cas5 and the large subunit, which is Cas8 or Cas10 in type I and III systems, respectively [25–29]. Typically, the C-terminus of the large subunit and multiple copies of Cas11, also known as the small subunit, runs along the belly of the complex as the minor filament [25,30].

### Type I system classification and mechanism

Type I systems, subtypes I-A through I-G, are characterised by the presence of the signature gene *cas3* (Figure 2A) [7]. Type I systems use an interference complex, termed Cascade (CRISPR-associated complex for antiviral defence), that recognises a protospacer adjacent motif (PAM) and targets dsDNA [31–33]. The PAM is a two to five base pair motif present on the invading dsDNA, but not in the CRISPR array, that allows Cascade to discriminate between ‘self’ and ‘non-self’ sequences [34,35]. The PAM is identified by the PAM recognition pocket in the large subunit, Cas8, which makes a combination of sequence- and structure-based interactions. Following PAM detection, the crRNA binds to the complementary target sequence and forms an R-loop in the dsDNA target [36,37]. For Cascade to fully bind the target, the first 8–10 nt (except position 6), which define the seed sequence of the crRNA, must have complete complementarity to the protospacer [36,38]. The R-loop displaces the non-target strand of foreign dsDNA and permits the crRNA to fully hybridise with the target strand [39,40]. The binding of the protospacer to the entire length of the crRNA induces conformational changes in the complex that lead to the recruitment of Cas3 [39,41]. Cas3 typically contains a metal-dependent histidine-aspartate (HD) nuclease domain (Cas3″) and a helicase domain (Cas3′) [42]. Cas3 nicks the non-target strand and generates a free end of ssDNA, which is then threaded through the helicase domain and is processively unwound via 3′–5′ helicase activity and degraded [37,39,42,43]. Targeting and degradation of invading DNA protects the host cell from viruses that may result in cell lysis and plasmids that contain potentially detrimental genes [44].

### Type III system classification and mechanism

Type III systems share a common interference complex architecture to type I systems; however, the targeted nucleic acids and mechanism of interference differ [14,45–48]. All type III CRISPR–Cas systems have multi-protein interference complexes that are evolutionarily related to type I Cascade and feature Cas protein homologues (Csm in types III-A/D/E/F and Cmr in types III-B/C), except the recently discovered type III-E system (also known as gRAMP and Cas7–11) that involves a single protein with multiple domains [16]. Type III CRISPR–Cas systems are defined by the signature gene *cas10* (Figure 2A). The Cas10 large subunit features an N-terminal HD nuclease domain and two polymerase-like cyclic (palm) domains, although only one site appears active [4]. Type III interference complexes bind to target RNAs that are complementary to their crRNA and have a unique mechanism to distinguish self from non-self targets. These type III interference complexes detect non-self-transcripts by recognising mismatches between the repeat-derived 5′ handle of the crRNA and the 3′ protospacer flanking sequence (PFS) of the target RNA. If complementarity between these sequences is detected — such as when antisense transcripts derived from CRISPR arrays are produced — Cas10 HD nuclease and cyclase activity is inactivated to prevent autoimmunity [47,49,50]. The detection of non-complementarity between the crRNA 5′ handle and the 3′ PFS of the target RNA license a conformational change and activation of Cas10 [49]. The Cas10 HD domain non-specifically degrades ssDNA, and the active palm domain catalyses the conversion of ATP to a range of cyclic oligonucleotides (cOAs) [47,48,50,51]. Cyclic oligoadenylates act as secondary messengers that allosterically activate ancillary nucleases that non-specifically degrade foreign and host-derived DNA/RNA and can induce host cell dormancy or death [47,48,52–55]. In addition, catalytically active RNA Recognition Motif (RRM) domains within the Cas7 backbone cleave the mRNA target at six nucleotide intervals [28,56,57]. The cleavage and release of the RNA fragments return the Cas10 HD and cyclase domains to their inactive state [58]. Targeting RNA by type III systems allows specific targeting of transcriptionally active plasmids and viral genomes [46,59].

## The type I-D CRISPR-Cas system

### Classification of the type I-D system

**Figure 3.**
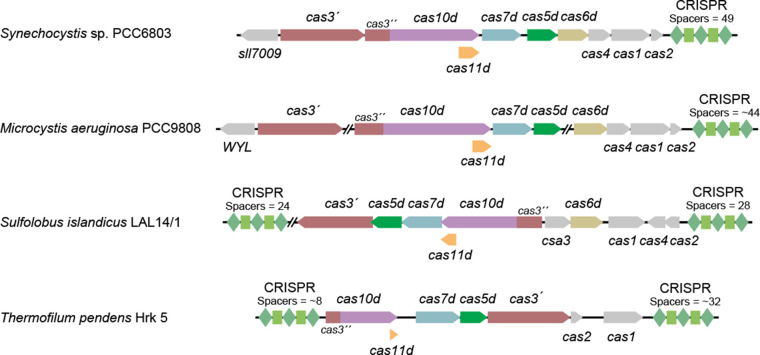
Schematic of representative type I-D *cas* genes and CRISPR arrays. CRISPR repeats are indicated by green diamonds, and spacers are indicated by green boxes. CRISPR arrays represent the position in locus, and the length is not correlated to the actual size of the CRISPR array.

The type I-D CRISPR–Cas system is abundant in archaea and cyanobacteria [4,24,60,61]. Type I-D is a hybrid system that contains signature genes of both type I and type III systems, *cas3* and *cas10*, respectively (Figure 3) [7]. However, *cas3* is split into its helicase (*cas3*′) and nuclease (*cas3*′′) domains, with the nuclease domain fused to *cas10* containing an inactivated palm domain*,* forming the large subunit *cas10d* (also known as *csc3*) [24]. Although the domain organisation of Cas10d resembles Cas10 with an HD nuclease domain at the N-terminus, this domain in Cas10d lacks the circular permutation of the histidine residues that is the hallmark of the type III Cas10 HD domain, and instead is reminiscent of the HD domain from other type I Cas3 proteins [4,17,24]. While the type I-D locus can vary between strains, it typically also contains type I-D orthologs of *cas1, cas2*, *cas4*, *cas5* (*cas5d*; *csc1*), *cas6*, (*cas6d*) *cas7* (*cas7d*; *csc2*)*,* and frequently a transcriptional regulator and CRISPR array(s) [7,19]. Additionally, within *cas10d* is an internal sequence (designated *cas11d*; Figure 3*)* that encodes the small subunit Cas11d [7,19]. Current studies have primarily used type I-D systems derived from several archaea and bacteria, notably the cyanobacterial strain *Synechocystis* sp. PCC6803 (Hereafter *Synechocystis*) (Figure 3). Some investigated systems appear to lack genes, such as *cas2* in *Sulfolobus islandicus,* and *cas4* and *cas6* in *Thermofilum pendens* (Figure 3). The lack of some genes may indicate these systems are inactive or may expropriate proteins or crRNA from other CRISPR–Cas systems in the host [4,60]. One example of cross-talk between CRISPR–Cas systems is the co-option of type I-F crRNAs by the type III-B system in *Marinomonas mediterranea* [62].

### Type I-D naïve adaptation

There are two types of adaptation in CRISPR–Cas systems, naïve and priming [8,63–65]. Naïve adaptation occurs when the adaptation complex establishes immunity against nucleic acids which have not been previously encountered [63]. Primed adaptation can occur when the interference complex associates with escape mutants or even during the normal processes of interference [65–67]. During primed adaptation, the interference complex interacts with the adaptation complex and allows the rapid acquisition of new spacers to bolster or restore immunity. Since there is currently no data on priming by type I-D systems, we will limit our discussion to naïve adaptation.

**Figure 4.**
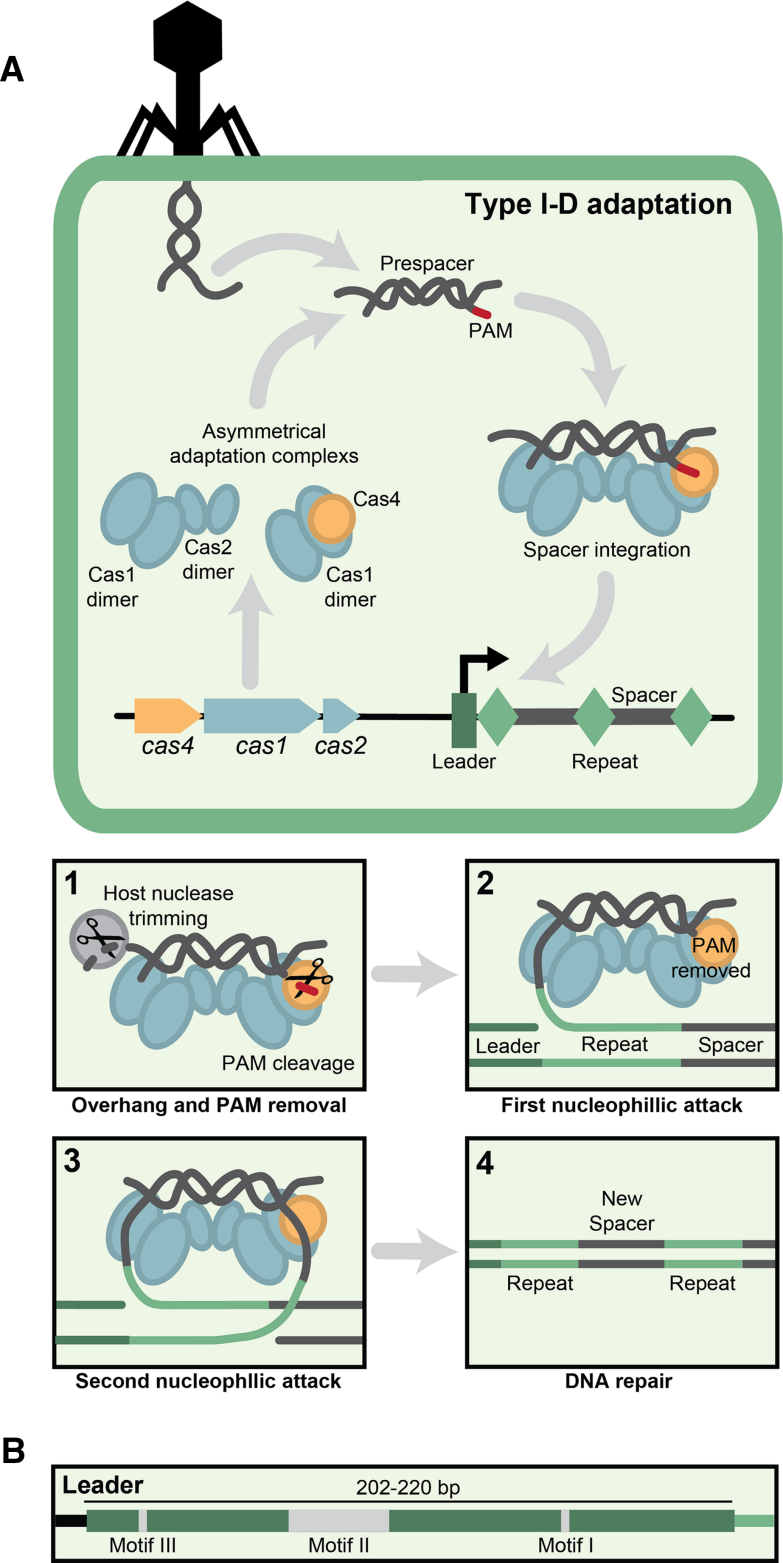
Predicted model of naïve type I-D adaptation. (**A**) The adaptation Cas proteins form asymmetrical complexes Cas1_2_:Cas2_2_ and Cas1_2_:Cas4_1_, which capture a prespacer. The PAM end is removed by Cas4, and opposite 3′ overhang is trimmed by a host nuclease (**1**). Half-site integration occurs via nucleophilic attack of the prespacer into the leader-repeat junction (**2**). Full-site integration occurs via nucleophilic attack of the second strand of the prespacer into the repeat-spacer junction (**3**). DNA repair completes integration to form a repeat-spacer-repeat at the leader proximal end of the array (**4**). Adapted from the model proposed by Kieper et al. [70]. (**B**) Schematic of the *Synechocystis* leader sequence and associated motifs.

The mechanism of type I-D adaptation has been determined predominantly through experiments using the *Synechocysti*s type I-D adaptation genes in the heterologous host *Escherichia coli* [68–70]. The nucleic acid source of the type I-D adaptation complex are fragments of dsDNA produced by DNA repair enzymes, and there is no orientation bias to a particular strand [68,71]. Adaptation by type I-D involves three proteins; Cas1, Cas2, and Cas4 (Figure 4A). Cas1 has nuclease and integrase domains [72], while Cas2 generally plays a structural role [72], and Cas4 has a PD-(D/E)XK RecB endonuclease domain [73,74]. During type I-D adaptation, two asymmetrical complexes exist within the cell to process the prespacer, Cas1_2_:Cas4_1_ and Cas1_2_:Cas2_2_ [70]. These asymmetrical complexes differ from other type I systems which typically form a single heterohexameric Cas1_4_:Cas2_2_ complex and can contain one to two Cas4 subunits [9,75]. The type I-D Cas1_2_:Cas4_1_ complex identifies and binds the PAM-containing 3′ overhang regions of DNA fragments, while the Cas1_2_:Cas2_2_ complex binds the opposite non-PAM containing 3′ overhang end [70,76]. While Cas1 and Cas2 are sufficient to capture and integrate spacers, Cas4 allows for the enrichment of spacers containing PAMs that support interference [68]. When the complexes have captured the dsDNA, Cas4 endonuclease activity is required to remove the PAM, leaving an overhang that is typically 6 nt in length [70]. Single nucleotide slipping can occur during PAM cleavage, which may play a role in primed adaptation [70,77]. It is unknown if the PAM recognition domain is present in Cas1 or Cas4, but the PAM sensing C-terminal tail in type I-E Cas1 is not conserved in type I-D Cas1 [70,78]. The non-PAM containing 3′ overhang is processed by an unknown endogenous nuclease [68,70].

The leader region of the CRISPR array is an A-T-rich region important for adaptation and contains the promoter for array expression [79]. The type I-D leader sequence in cyanobacteria is 202–220 bp, which is longer than other systems, such as type I-E, which is typically less than 100 bp (Figure 4B) [69]. Within the type I-D leader sequence, three conserved motifs enhance spacer uptake: motifs I, II, and III [69]. While the exact function of each motif is yet to be identified, Kieper et al. [69] noted that motif III resembled the integrase anchoring site in type I-E system, a sequence that interacts with the adaptation machinery upon DNA bending by Integration Host Factor (IHF). However, as there is no homologue of IHF in *Synechocysti*s, the authors speculated that another DNA-binding host factor may bind at motif II and facilitate DNA bending to position motif III in proximity of the adaptation complex [69,80]. Kieper et al. [70] proposed a model (Figure 4A) in which the type I-D adaptation complex sequence-specifically identifies the leader region and positions the Cas1_2_:Cas2_2_ complex to catalyse half-site integration of the non-PAM end into the leader proximal end of the repeat. The Cas1_2_:Cas4_1_ complex is predicted to pause to allow the correct orientation of the spacer within the CRISPR array. The Cas1_2_:Cas4_1_ complex next catalyses the integration of the end that previously contained the PAM into the downstream repeat-spacer junction. Endogenous DNA repair systems then repair the repeat regions, resulting in the new spacer being fully integrated between two repeats into the leader-proximal region of the CRISPR array [70,81]. Spacers integrated into the type I-D system CRISPR array were most frequently 35 nt in length; however, the size varied by 5 to 6 nt, which is common in other systems containing Cas4 [68,82]. When Cas4 was removed from the system, the spacer size increased by 1 nt to 36 nt, indicating that Cas4 plays a role in correct spacer processing [68].

### Type I-D expression and processing

**Figure 5.**
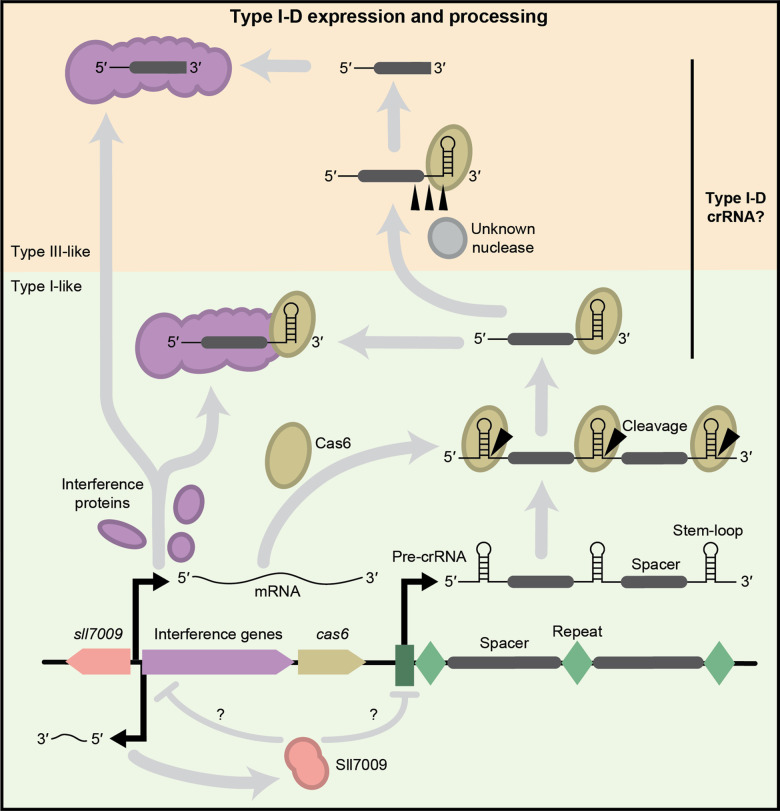
Predicted model of type I-D expression and processing. The *cas* (purple and brown) and regulator (pink) genes are transcribed and translated while the CRISPR array is transcribed, forming the pre-crRNA. Sll7009 may regulate type I-D interference via any of the promoters. Cas6 associates with the stem–loops of the pre-crRNA and cleaves at the base of the stem, forming type I-like crRNA (green background), as observed by McBride et al. [19]. Shorter type I-D crRNA also occurs (Scholz et al. [83]), resembling type III-like mature crRNA (yellow background).

Before type I-D Cascade can form, *cas* genes and crRNA are expressed. The type I-D interference genes *cas3′*, *cas5d*, *cas6d*, *cas7d*, and *cas10d* are transcribed and then translated (Figure 5) [83]. The small subunit of the type I-D system, Cas11d, is expressed from an in-frame alternative translational start site within the 3′ end of *cas10d* [19]. This phenomenon is conserved across type I-D, I-B, and I-C systems [17,19,23].

The expression and processing of type I-D crRNAs have primarily been studied in *Synechocystis* [19,83]. Like other type I systems, the CRISPR array is expressed as a pre-crRNA, and the palindromic repeats present in each repeat form stem–loop secondary structures (Figure 5) [83–85]. Cas6d binds the pre-crRNA in a sequence- and secondary structure-dependent manner and cleaves the repeat sequence after the stem–loop to create an ∼72 nt crRNA with an 8 nt 5′ and a 29 nt 3′ repeat-derived handles [83,86]. This 72 nt crRNA was the predominant crRNA species present within type I-D Cascade when recombinantly expressed and purified from *E. coli* [19]. However, in the natural host *Synechocystis*, there appears further maturation to 45 and 39 nt [83]. Similar maturation of the *S. islandicus* type I-D crRNA was observed when extracted from type I-D Cascade expressed in *S. islandicus*, revealing cRNAs of 49 ± 1 nt [18]. In *Synechocystis*, the 6 nt difference in mature crRNA lengths indicates step-wise processing by a host nuclease(s), similar to type III systems and suggests complexes lacking Cas6 and a Cas7 subunit(s) [83,87]. The type I-D crRNA may have undergone maturation with unidentified endoribonucleases present within its native host, *Synechocystis*, but not in an *E. coli* heterologous host.

Type I-D systems frequently co-occur with a DNA binding regulator that likely responds to signal(s) to elicit changes in CRISPR–Cas activity (Figure 3) [4,60]. In *Synechocystis*, this regulator is called Sll7009 and contains a WYL (tryptophan, tyrosine, leucine) motif that is predicted to bind small negatively charged ligands and a winged helix-turn-helix domain that binds DNA [88–90]. Regulators are associated with other type I-D systems, including the WYL-containing protein from *M. aeruginosa* and Csa3 from *S. islandicus,* which contains a CRISPR-associated Rossman fold (CARF) domain. The *Synechocystis* type I-D system includes three promoters, one that controls the *cas* genes, a second that controls the CRISPR array, and a third that controls the Sll7009 regulator (Figure 5) [91]. Hein and colleagues identified Sll7009 as a negative regulator of the type I-D system by knocking out *sll7009* and observing an increase in mature crRNA [90]. Sll7009 likely regulates the type I-D system by coupling cell signals with the repression of at least one of the type I-D promoters.

### Type I-D interference

**Figure 6.**
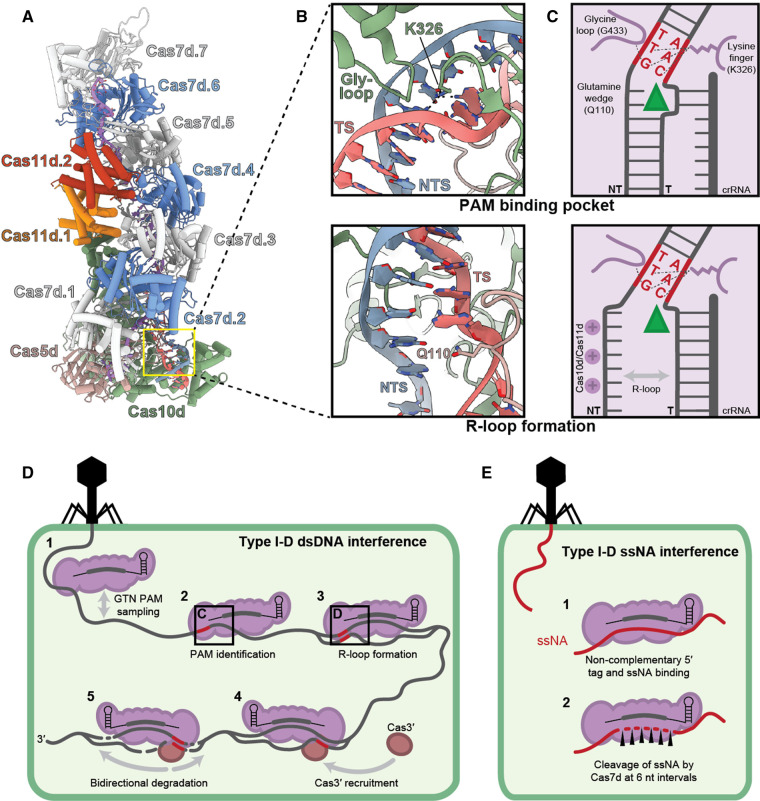
Model of type I-D interference for dsDNA and single-stranded nucleic acids (ssNA). (**A**) Structure of *Synechocystis* type I-D Cascade (Protein Data Bank ID, 7SBA). Subunits displayed: Cas10d (green); Cas5d (pink); Cas11d (orange); Cas7d (grey and blue); crRNA (purple); target strand (TS; red); non-target strand (NTS; cyan). Structure (**B**) and schematic (**C**) of the PAM binding pocket, including the glycine loop and lysine finger from Cas10d (top) and the glutamine wedge from Cas5d positioned where dsDNA bifurcation occurs (bottom). R-loop formation is likely stabilised by a positive patch within Cas10d and Cas11d. (**C**) adapted from Hayes et al. [94]. (**D**) The predicted mechanism of type I-D interference of dsDNA. Type I-D Cascade samples dsDNA looking for a GTN PAM (**1**), the PAM is identified with the PAM binding pocket in Cas10d (**2**), and an R-loop is formed (**3**), Cas3′ is recruited to Cascade (**4**), bidirectional degradation of both strands occurs (**5**). (**E**) The predicted mechanism of type I-D interference of single-stranded nucleic acids (ssNA). Type I-D Cascade binds the ssNA (**1**), followed by the cleavage of the ssNA by Cas7d (**2**).

The type I-D system has been demonstrated to be an active and functional system within *Synechocystis* [68]. The *Synechocystis* type I-D system uses a 5′-GTN-3′ interference PAM *in vivo* and *in vitro*, where ‘N’ stands for any nucleotide [18,19,68]. The *M. aeruginosa* interference PAM differs slightly, with a 5′-GTH-3′ PAM, where ‘H’ can be A, C, or T [21]. Insight into the interference mechanism has been gained by high-resolution structures of the *Synechocystis* type I-D Cascade and the Cas10d subunit bound to an anti-CRISPR protein from *S. islandicus* [17,92]. Type I-D Cascade has a helical major filament composed of multiple Cas7d subunits (Figure 6A). Cas6d likely binds the stem region at the 3′ end of the crRNA; however, despite Cas6d being co-purified with the complex, it was not resolved in the structure [17]. The 5′ end of the crRNA is capped with Cas5d and Cas10d. The C-terminus of Cas10d and the alternatively translated Cas11d subunits run along the belly of the complex, forming the minor filament [17,19]. The overall architecture of type I-D Cascade better resembles type III complexes rather than type I Cascades, particularly as the angular trajectory of the crRNA is consistent with type III crRNA [17,19].

Type I-D Cascade is capable of binding dsDNA [19], ssDNA [17,18], and ssRNA [17] with high affinity. During dsDNA interference, Cascade randomly samples dsDNA to identify a PAM (Figure 6B–D) [18,32,68,93]. The most well-studied type I system is type I-E, which uses three elements within the PAM binding pocket in the large subunit to detect the PAM: a glycine-rich loop, a lysine finger, and a glutamine wedge [94]. The glycine-rich loop is inserted into the minor groove of the DNA and results in DNA bending; both the glycine-rich loop and the lysine finger directly interact with the nucleotides to determine their specificity. The glutamine wedge is inserted after the PAM and causes bifurcation of the dsDNA [94,95]. PAM detection by type I-D Cascade was elucidated with the structure of the *Synechocystis* type I-D Cascade bound to a 5′-GTT-3′ PAM-protospacer [17]. The PAM is identified by the PAM recognition domain within Cas10d. A glycine-rich loop from the PAM recognition domain is inserted into the minor groove of the dsDNA, where a glycine (G433) interacts with the guanine nucleotide on the non-target strand in the −3 PAM position (G_NTS-3_) (Figure 6B,C). The lysine finger (K326) in the PAM recognition domain interacts with nucleotides C_TS-3_, T_NTS-2_, and G_NTS-3_. These specific interactions of Cas10d with the −2 and −3 positions of the PAM are consistent with the third nucleotide being flexible as ‘N’ in GTN. The dsDNA is split directly after the PAM by a cleft composed of the bottom of Cas7d and Cas10d. Type I-D Cascade does not possess a conventional glutamine wedge from the large subunit; however, a Cas5 glutamine residue (Q110) inserts between the dsDNA where the strands separate, which may constitute the type I-D glutamine wedge (Figure 6B).

The bifurcation of the dsDNA allows the target strand to hybridise with the complementary crRNA in Cascade and displaces the non-target strand to form an asymmetrical R-loop [17]. When the crRNA binds the protospacer, every sixth base, starting at position one, is flipped out by the thumb domain of the Cas7 subunits, allowing for mismatches at these sites [17,22,96]. The recognition of the PAM and the protospacer causes a conformational shift of the Cas3″ and Cas11d domains of Cas10d and the Cas11d subunits [17]. This conformational change may support the stabilisation of the R-loop through interactions of the non-target strand with positively charged residues in the C-terminus of Cas10d and Cas11d subunits [17,19] and direct the non-target strand towards the Cas10d active site. The Cas3″ domain of Cas10d has a type I-like HD active site responsible for target cleavage [17,18,22,97]. Cas3′ has been shown to interact with Cas10d [22], and this HD domain is likely where the Cas3′ helicase docks [17]. Upon the recruitment of Cas3′, Cas3″ nicks the non-target strand and, in an ATP-dependent manner, nicks further sequences upstream of the protospacer on the non-target strand and both sides of the protospacer on the target strand [18]. The observed bidirectional cleavage upstream of the protospacer is consistent with the helicase-nuclease activity of Cas3′–Cas10d on each strand after the initial cleavage at the protospacer region. While most type I systems display unidirectional cleavage [23,98,99], the type I-A Cascade from *Pyrococcus furiosus* was also recently shown to cleave in a bidirectional manner [100]. Interestingly, types I-A and I-D both have split *cas3* helicase and nuclease genes [24], suggesting a separately encoded helicase might be important for bidirectional cleavage. A similar model of two helicases operating on different DNA strands was recently proposed for type IV-A interference, where helicase CasDinG facilitated bidirectional DNA depletion [101]. The precise molecular details of how bidirectional cleavage occurs are still emerging and further characterisation is required.

Type I-D Cascade can also bind single-stranded DNA and RNA *in vitro* [17,18]. Type I-D Cascade bound ssDNA and RNA with a 5′-AAC-3′ PFS, complementary to a 5′-GTT-3′ PAM, and a scrambled PFS (5′-ACG-3′) [17], indicating single-stranded binding by type I-D Cascade appears to have a broader range of PFS requirements and different from those required for dsDNA binding [17,19,68]. Furthermore, when type I-D Cascade is bound to ssRNA, the PAM recognition domain becomes flexible, in contrast with the rigid form bound to dsDNA [17]. When type III systems bind target RNA, the palm domain of Cas10 converts ATP to cOA signalling molecules, and the Cas7 subunits cleave the target [28,47,48]. Type I-D is not expected to produce cOA molecules as the palm domain is predicted to be inactive in Cas10d [7]. Unexpectedly, the Cas10d subunit from *Microcystis aeruginosa* PCC9808 was recently shown to have ATPase activity; however, the role of this function is unknown [22]. The Cas7 subunits in Cascade have both sequence and structural homology to their active type III counterpart [24,96]. When type I-D Cascade is bound to ssDNA, the active Cas7 subunit cleaves the ssDNA at 6 nt intervals (Figure 6E) [18], similar to the cleavage of RNA in type III systems [28,56]. However, the exact mechanism of ssDNA cleavage and which residues are involved is unknown [18].

Type I-D Cascade can bind dsDNA [18,19], ssDNA [17,18], and ssRNA [17] in a sequence-specific manner, which may indicate that the type I-D system is a broad defence mechanism. However, *in vivo* interference has only been tested and demonstrated against dsDNA targets [21–23,68]. Interestingly, type I-D Cascade appears to have a binding preference for single-stranded nucleic acids over double-stranded ones, based on the binding affinity *in vitro* [17,19]. This preference may reflect the type of bacteriophage (phage) or stage of phage replication that type I-D Cascade targets. For example, if type I-D Cascade does target ssRNA *in vivo*, it may target single-stranded RNA phages or phage mRNAs produced during transcription. Alternatively, if type I-D does target ssDNA *in vivo*, it may be an ssDNA phage or ssDNA exposed during replication or transcription of the phage genome.

### Viral evasion mechanisms of the type I-D system

Viruses have evolved mechanisms to counteract prokaryotic immune mechanisms, allowing for phage propagation [2,5]. To evade CRISPR–Cas immunity, phages can acquire mutations that escape detection, use anti-CRISPR proteins or protect their DNA within a nucleus-like structure [102–104]. Anti-CRISPR proteins inactivate CRISPR–Cas immunity via diverse mechanisms [44]. The only known anti-CRISPR for the type I-D system is AcrID1 (Anti-CRISPR against type I-D) from the virus SIRV3 that infects *S. islandicus* [92,97]. AcrID1 is a 13 kDa protein that forms a homodimer with a net negative charge [97]. This dimer interacts directly with Cas10d, contacting all domains within Cas10d [92,97]. Addition of AcrID1 inhibited type I-D Cascade from degrading dsDNA [97]. Manav and colleagues suggested that two AcrID1 dimers sequester two Cas10d subunits forming an AcrID1_4_:Cas10d_2_ complex, which would prevent Cas10d from complexing with the other Cas proteins to form a functional Cascade. Another potential inhibition method by AcrID1 was proposed by Schwartz and colleagues, who modelled AcrID1 bound to the positively charged belly of Cas10d in Cascade [17]. In this model, AcrID1 was hypothesised to obstruct the path of the non-target strand toward the Cas10d HD domain; therefore, preventing R-loop formation, subsequent Cas3′ recruitment and target degradation.

### Type I-D system as an evolutionary intermediate

**Figure 7.**
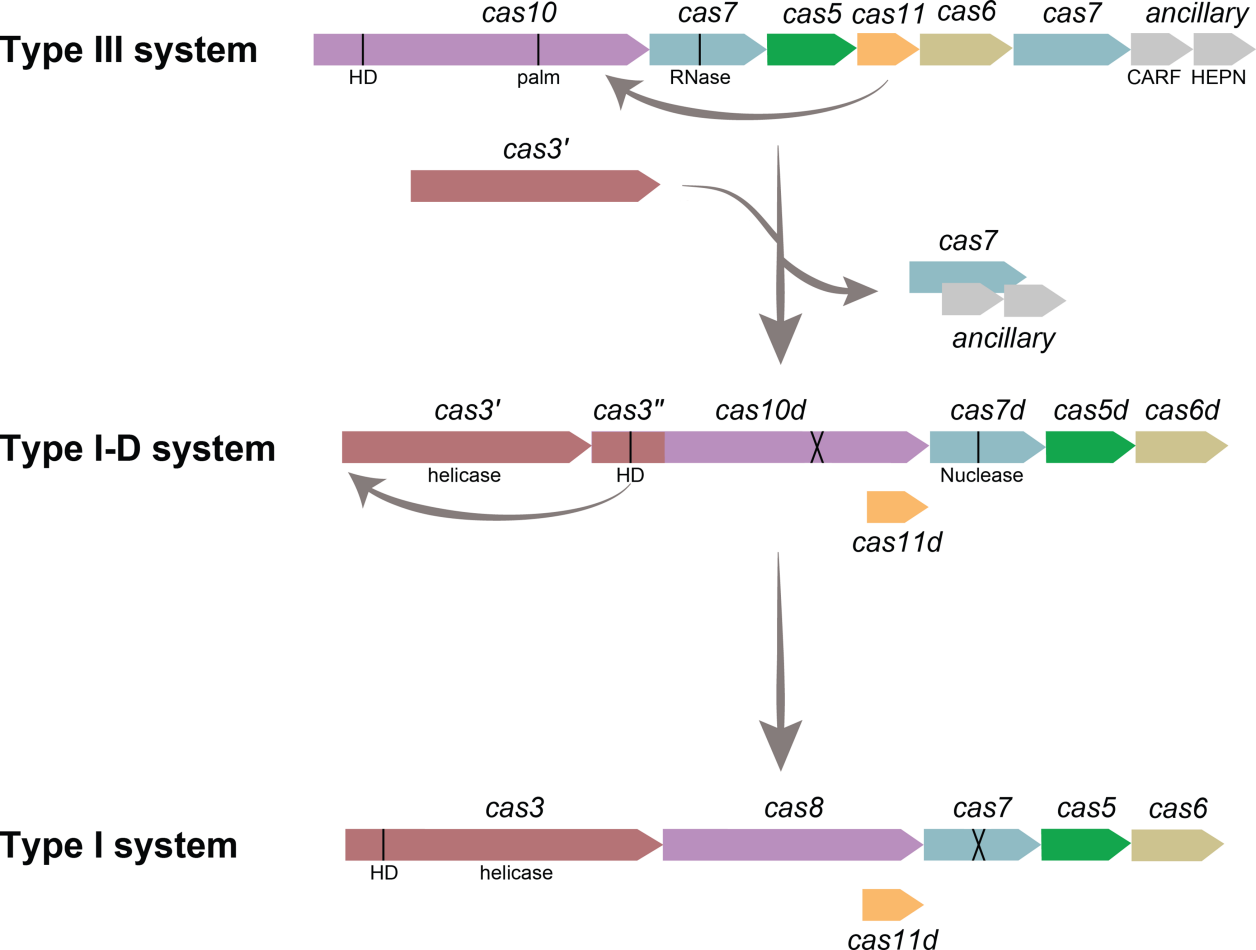
Predicted model of the type I-D system as an evolutionary intermediate between type III and type I systems. Type I-D likely evolved from a type III-like ancestor system through the acquisition of a helicase domain, the loss of the circular permutation in the *cas10d* HD, *cas11* becoming alternatively expressed from the large subunit, and the loss of one *cas7* and the auxiliary genes. Type I systems may have evolved from type I-D via the transfer of the HD domain, *cas3″*, onto the helicase domain, *cas3′*, to form the typical *cas3* nuclease-helicase. In some type I systems, duplication of *cas11* occurs*,* forming a separate gene. A black line indicates key domains, and inactive domains are indicated by a cross.

Type I-D CRISPR–Cas systems are frequently found in archaea and cyanobacteria [24,60,105]. Phylogenetic analysis of the Cas10d subunit indicated four lineages represented by two cyanobacterial groups, one with predominantly other bacteria, and one with mostly archaea [7,19]. Across all groups, the internal expression of Cas11d appeared widely conserved. The features of type I-D systems that are reminiscent of type I and III systems have led to it being a predicted evolutionary intermediate [7,17–19]. Combined with a CRISPR–Cas origin model proposed by Koonin and Makarova [106], these observations allow for the proposal of a model for the evolution of type I-D systems (Figure 7).

The type I-D system was likely formed from a type III-like ancestor through the inactivation of the cyclase palm domain within *cas10,* the loss of associated ancillary nucleases and a *cas7* gene (Figure 7) [7,24]. The ancestral system then acquired the helicase, *cas3*′. The circular permutation of histidines within the *cas10* HD domain was lost, forming a type I-like HD domain, *cas3″* [106]. The type I-D Cas7d subunit has nuclease activity, comparable to the type III Cas7 but in contrast with other type I Cas7 subunits [7,16,18,96]. From the type III-like ancestor to type I-D, the *cas11d* gene appears to fuse onto the end of *cas10*, retaining its translational start site [19]. Comparison of type I-D systems from different genomes revealed that the genes from the effector module were shuffled and the adaptation module genes, and/or *cas6,* remained conserved [106,107]. We compared diverse type I-D systems in genomes from cyanobacteria, other bacteria and archaea phyla groups [19] and found the gene order of the effector module differed between these groups, with notable differences with the associated regulator and the positions of *cas3′* and *cas6* (Supplementary Figure S1).

A model for the evolution of type I systems from type I-D begins with the HD domain, *cas3*′′, being transferred onto the helicase *cas3*′, resulting in the evolution of a smaller, enzymatically inactive large subunit (*cas8*) [106]. The alternative translational start site for *cas11* was retained in some systems and may have been duplicated to become a separate gene in others. However, the evolutionary lineage of Cas11 in type I systems is unknown, and further analysis is required [19]. The Cas7d nuclease activity was lost, which resulted in inactive Cas7 subunits within other type I systems [17,106]. Further sequence and structural analysis of type I and III systems may provide further insight into the evolutionary steps that formed class 1 systems.

### Biotechnological applications of type I-D systems

Over the past decade, many CRISPR–Cas systems have been co-opted as sequence-specific genome engineering technologies, which have sparked significant advances in fundamental science, precision medicine, and biotechnology [20]. Initial CRISPR–Cas technologies were developed using class 2 CRISPR–Cas systems, such as Cas9 and Cas12, which have single multi-domain effector proteins that can be programmed to generate double-stranded breaks within/adjacent to the target site [108,109]. The class 2 systems are easily engineered, smaller than class 1 systems, and amenable for use across heterologous host species [109–112]. In contrast, the more abundant type I CRISPR–Cas systems are underutilised as biotechnological tools yet possess unique attributes that are attractive for exploitation [113]. Cas3-mediated processive degradation introduces long-range deletions into the target genome – a distinct function that has been harnessed to develop a new suite of CRISPR–Cas tools [113]. For example, the type I-E system was used to make large deletions at endogenous targeted loci in human cells [98]. Furthermore, given the natural abundance of type I systems in clinically and industrially relevant bacteria, these endogenous systems can be harnessed for applications such as precision antimicrobials and strain engineering [99,114–117]. Another advantage of harnessing type I systems is their diverse PAM requirements that differ from those required by Cas9 and Cas12-based tools, which expands the range of available target sites [32]. The newly characterised type I-D system is among the most recent additions to the type I CRISPR–Cas toolbox and, thus far, has been developed as a gene editing platform [21]. As a hybrid between a type I and III system, the type I-D system has distinct structural and functional attributes that display potential for further exploitation in developing programmable molecular tools [17–19].

The type I-D system is among several type I CRISPR–Cas effectors that have been converted into sequence-specific genome editing technologies [21–23,98,100,118–120]. The exploitation of type I CRISPR–Cas systems to generate deletions was originally performed by Vercoe *et al*., who used an endogenous type I-F system to isolate mutants with 30–100 kb deletions in the core and accessory genome of *Pectobacterium atrosepticum* [99]. In addition, several type I-E systems have been shown to induce similar long-range chromosomal (30–100 kb) deletions in human cells [98,99,118,119]. In all type I-E editors studied, the Cas3-generated deletions were limited to regions upstream of the PAM and target site. Subsequent studies of other type I editors showed the type I-B and I-C systems also generated long-range unidirectional deletions upstream of the PAM when applied in human cells [23].

Recently, the type I-D system from *M. aeruginosa* was adapted for gene editing of human and plant genomes [21,22]. The type I-D system generated a spectrum of bidirectional long-range chromosomal deletions from 2.5 to 19 kb with ≤57% editing efficiency in both eukaryotic cell types — a pattern consistent with type I-D *in vitro* cleavage pattern [18]. The type I-D complex also introduced double-stranded breaks and small deletions (1–12 bp) within the target site at up to 19% editing efficiency [22]. Interestingly, the type I-A system also produced bidirectional deletions in human cells that spanned up to 2.2 kb [100]. Notably, unidirectional Cas3-induced deletions in other systems (types I-B, -C and -E) appear longer (≤100 kb) than their bidirectional counterparts (≤19 kb) [21–23,98,100,118,119]. The disparity in length between the two types of deletion may be due to the unidirectional deletions preserving the PAM and protospacer and allowing multiple rounds of editing [118]. In contrast, the bidirectional deletions induced by types I-A and I-D destroyed the PAM and target site, preventing multiple rounds of editing [21,22,100]. Further characterisation of Cas3-driven interference mechanisms will provide insight into how CRISPR–Cas type I systems can be further exploited for gene editing technologies.

As previously discussed, diverse type I CRISPR–Cas complexes require the independent translation of the small subunit Cas11 from an alternative start site located within the large subunit, including Cas11d from within *cas10d* of the type I-D system [19]. The internal translation of Cas11 is a critical consideration when implementing these specific type I systems for biotechnological applications in eukaryotes due to translational machinery differing from their natural prokaryotic hosts. As predicted by McBride et al. [19] the reconstitution of specific type I-B, I-C, and I-D systems in eukaryotic cells may require *cas11* encoded from a separate gene. Tan and colleagues recently confirmed these predictions with gene editing studies using examples of type I-B, I-C, and I-D systems, including the *Synechocystis* type I-D system. In all cases, gene editing improved when Cas11 was separately expressed, including in *Synechocystis* type I-D, which achieved 5% editing efficiency with Cas11 but had 0% efficiency without [23]. Combined with the role of Cas11 in dsDNA binding, the decreased editing efficiency is predicted to be caused by a dramatic decrease in dsDNA binding capability [19,23]. Interestingly, Osakabe and colleagues reported that the type I-D system from *M. aeruginosa* could introduce gene edits in human cells without the need for separately encoded Cas11d. However, it was unclear from these studies whether Cas11 was expressed at sufficient levels to maximise editing efficiency [21].

Until recently, type I-D systems were largely uncharacterised; therefore, the full spectrum of biotechnological applications for these systems is still emerging. Like other type I systems, the type I-D system could be utilised for endogenous chromosomal targeting applications such as selective killing and strain engineering [99,120–122]. The type I-D complex could also be engineered for programmable gene regulation, as has previously been achieved with catalytically inactivated type I-E and I-F systems [20,123–126]. Recent studies of type I-D systems from *S. islandicus* and *Synechocystis* have shown that the hybrid features of type I-D complexes allow the binding of dsDNA, ssDNA and ssRNA [17–19]. Further studies into how these nucleic-acid targeting activities are co-ordinated during type I-D interference may reveal a suite of complementary biotechnological applications that will further expand the CRISPR–Cas toolkit.

## Concluding remarks

The type I-D CRISPR–Cas system displays genetic, structural, and mechanistic aspects of both type I and type III CRISPR–Cas systems and are often referred to as chimeric [7,17–19]. The type I-D system is likely an evolutionary intermediate of class 1 CRISPR–Cas systems, where it evolved from a type III-like ancestor and later evolved into the ‘typical’ type I system [7,106]. The chimeric nature of the type I-D system led to research into its mechanisms of adaptation, expression and processing, and interference, and has further confirmed that the type I-D system displays features of both type I and III systems [7,17–19,68–70,83]. For example, the structure of the type I-D Cascade interference complex better resembles a type III complex, and it binds and cleaves substrates in a type I- and type III-like manner [7,17–19]. The unusual features of the type I-D CRISPR–Cas system also lends itself to exploitation as novel biotechnological tools, such as for gene editing [21–23,98,100,118–120]. Despite these recent advances, there are still many key questions (see Key considerations box) into the mechanism of type I-D systems. As new details emerge into how type I-D functions, new opportunities for biotechnological exploitation may arise.

**KEY CONSIDERATIONS**

- Does the type I-D system elicit primed adaptation, and if so, does it occur in a bidirectional manner?
- Is Cas6d part of the final Cascade complex *in vivo,* or is it removed during crRNA processing?
- How does the regulator associated with the type I-D system control expression and what signals does it respond to?
- What is the mechanism for bidirectional deletions by the type I-D system?
- Does the single-stranded nucleic acid targeting of the type I-D system have a physiological role?
- Does Cas11d provide new insight into the evolutionary lineage of class 1 CRISPR–Cas systems?

## Abbreviations

HD: histidine-aspartate
IHF: Integration Host Factor
PAM: protospacer adjacent motif
PFS: protospacer flanking sequence
CRISPR-Cas: clustered regularly interspaced short palindromic repeats and CRISPR-associated proteins
Cascade: CRISPR-associated complex for antiviral defence
MGE: mobile genetic element
