# Supplementary material for: The biology and type I/III hybrid nature of type I-D CRISPR–Cas systems
Source: Biochem J. 2023 Apr 13;480(7):471–88. doi: 10.1042/BCJ20220073 (PMC10212523; doi:10.1042/BCJ20220073)
Supplement: Supplementary Material [file BCJ-480-471-s1.pdf]

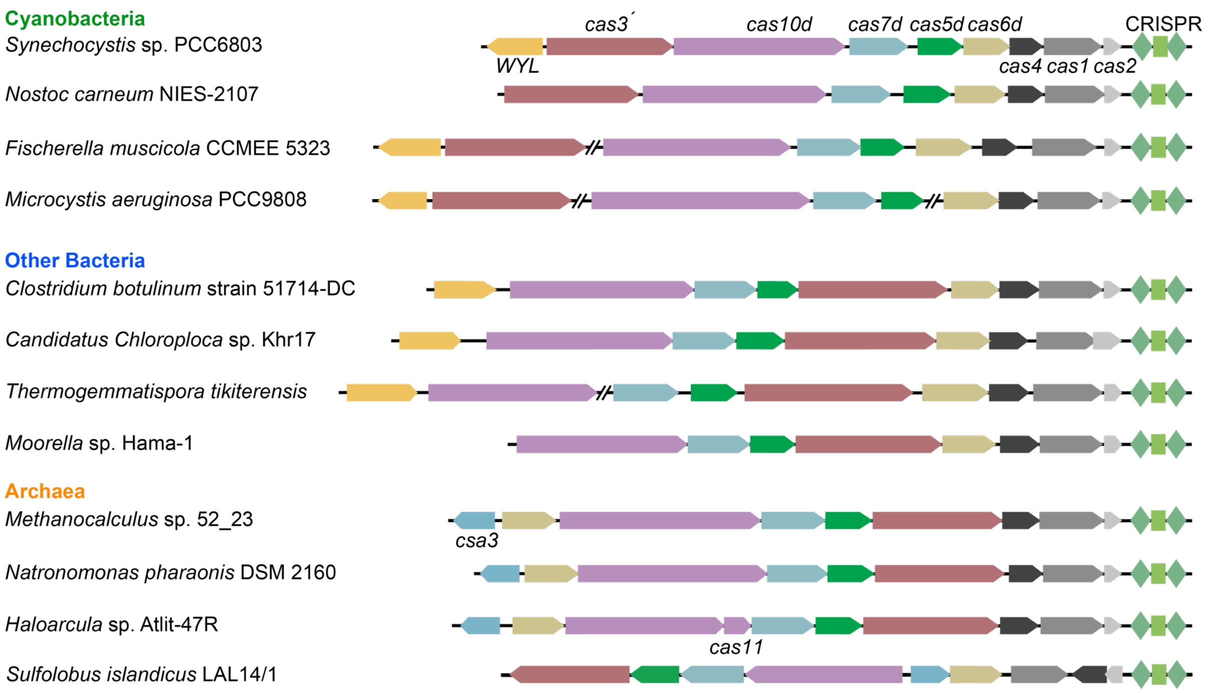

Supplementary Figure 1. Alternative gene arrangements occur in the type I-D CRISPR-Cas system according to phylogenetic group. The figure schematically shows selected CRISPR–*cas* loci of the type I-D CRISPR-Cas system grouped according to phylum. Homologous genes are colour-coded and identified by a family name. Internally expressed *cas11* annotations have been removed, although *Haloarcula* sp. Atilt-47R appears to have a separate *cas11* gene unlike other type I-D systems [7, 19]. Archaeon *Sulfolobus islandicus* LAL14/1 had distinct gene rearrangements compared to other systems, which is supported by previous phylogenetic analysis that indicated Cas10d from archaeon *Sulfolobus islandicus* LAL14/1 did not group with other archaea or bacteria [7, 19]. Note that only the CRISPR array directly adjacent to the adaptation genes is shown for each representative. Other arrays may be present nearby or elsewhere in these genomes.
